# Cardiac repair after myocardial infarction: A two-sided role of inflammation-mediated

**DOI:** 10.3389/fcvm.2022.1077290

**Published:** 2023-01-09

**Authors:** Tingting Li, Zhipeng Yan, Yajie Fan, Xinbiao Fan, Aolin Li, Zhongwen Qi, Junping Zhang

**Affiliations:** ^1^First Teaching Hospital of Tianjin University of Traditional Chinese Medicine, National Clinical Research Center for Chinese Medicine Acupuncture and Moxibustion, Tianjin, China; ^2^Graduate School, Tianjin University of Traditional Chinese Medicine, Tianjin, China; ^3^Xiyuan Hospital of China Academy of Chinese Medical Sciences, Beijing, China

**Keywords:** inflammation, myocardial infarction, cardiac repair, inflammatory cells, epigenetic modifications, two-way adjustment

## Abstract

Myocardial infarction is the leading cause of death and disability worldwide, and the development of new treatments can help reduce the size of myocardial infarction and prevent adverse cardiovascular events. Cardiac repair after myocardial infarction can effectively remove necrotic tissue, induce neovascularization, and ultimately replace granulation tissue. Cardiac inflammation is the primary determinant of whether beneficial cardiac repair occurs after myocardial infarction. Immune cells mediate inflammatory responses and play a dual role in injury and protection during cardiac repair. After myocardial infarction, genetic ablation or blocking of anti-inflammatory pathways is often harmful. However, enhancing endogenous anti-inflammatory pathways or blocking endogenous pro-inflammatory pathways may improve cardiac repair after myocardial infarction. A deficiency of neutrophils or monocytes does not improve overall cardiac function after myocardial infarction but worsens it and aggravates cardiac fibrosis. Several factors are critical in regulating inflammatory genes and immune cells’ phenotypes, including DNA methylation, histone modifications, and non-coding RNAs. Therefore, strict control and timely suppression of the inflammatory response, finding a balance between inflammatory cells, preventing excessive tissue degradation, and avoiding infarct expansion can effectively reduce the occurrence of adverse cardiovascular events after myocardial infarction. This article reviews the involvement of neutrophils, monocytes, macrophages, and regulatory T cells in cardiac repair after myocardial infarction. After myocardial infarction, neutrophils are the first to be recruited to the damaged site to engulf necrotic cell debris and secrete chemokines that enhance monocyte recruitment. Monocytes then infiltrate the infarct site and differentiate into macrophages and they release proteases and cytokines that are harmful to surviving myocardial cells in the pre-infarct period. As time progresses, apoptotic neutrophils are cleared, the recruitment of anti-inflammatory monocyte subsets, the polarization of macrophages toward the repair phenotype, and infiltration of regulatory T cells, which secrete anti-inflammatory factors that stimulate angiogenesis and granulation tissue formation for cardiac repair. We also explored how epigenetic modifications regulate the phenotype of inflammatory genes and immune cells to promote cardiac repair after myocardial infarction. This paper also elucidates the roles of alarmin S100A8/A9, secreted frizzled-related protein 1, and podoplanin in the inflammatory response and cardiac repair after myocardial infarction.

## Introduction

Myocardial infarction (MI) is one of the most prevalent and leading causes of death and disability throughout the world (1, 2). In MI, myocardium blood flow is suddenly reduced due to lumen occlusion caused by plaque rupture and thrombosis, which leads to pathological changes such as ischemia and hypoxia in the myocardial tissue. Myocardial tissue in the ischemic zone maintains the ejection fraction at the expense of enlarging the ventricular chamber. Still, the prolonged increase in ventricular wall pressure inevitably causes structural and functional changes in the ventricle’s infarcted and non-infarcted zones, ultimately leading to cardiac failure and death (3). Although thrombolysis, percutaneous coronary intervention (PCI), and coronary artery bypass grafting may reduce the rate of emergency hospitalization or mortality within 30 days of hospital discharge in patients with MI, this did not prevent long-term adverse cardiovascular events after MI. To prevent MI patients from developing cardiac failure, new treatments are needed to reduce the size of MI and improve cardiac function (4).

The inflammatory response is the primary pathological event in myocardial injury and determines the size of MI and subsequent adverse cardiac remodeling. Growing evidence suggests that the timely resolution of inflammatory processes may help prevent adverse cardiac remodeling and cardiac failure (5). The initial inflammatory response to MI results from stimulation by foreign pathogen-associated molecular patterns (PAMPs) and the release of damage-associated molecular patterns (DAMPs) from a large number of necrotic myocytes and extracellular matrix (ECM) fragments in the infarcted myocardium, which are early warning mediators of the body’s damage and are referred to as “danger signals” (6). The immune system senses these “danger signals” through a complex and sophisticated network of molecules that activate multiple inflammatory signaling pathways, including high mobility group box 1 (HMGB1), a key trigger of inflammatory injury after myocardial ischemia, cardiac heart shock protein (HSP) and low molecular hyaluronic acid, triggering an intense inflammatory response (7, 8). In addition, antioxidant mechanisms are disrupted after myocardial injury, generating large amounts of reactive oxygen species (ROS) and inducing signaling of inflammation is upregulated, further exacerbating the inflammatory response. The transduction of these inflammatory signals eventually leads to the activation of Toll-like receptors (TLRs) and nuclear factor kappa-B (NF-κB) membrane receptors, which promote the release of various inflammatory and chemokines, leading to the infiltration of large numbers of immune cells into the damaged tissue and removes necrotic cells and debris from the MI region (8).

**FIGURE 1:**
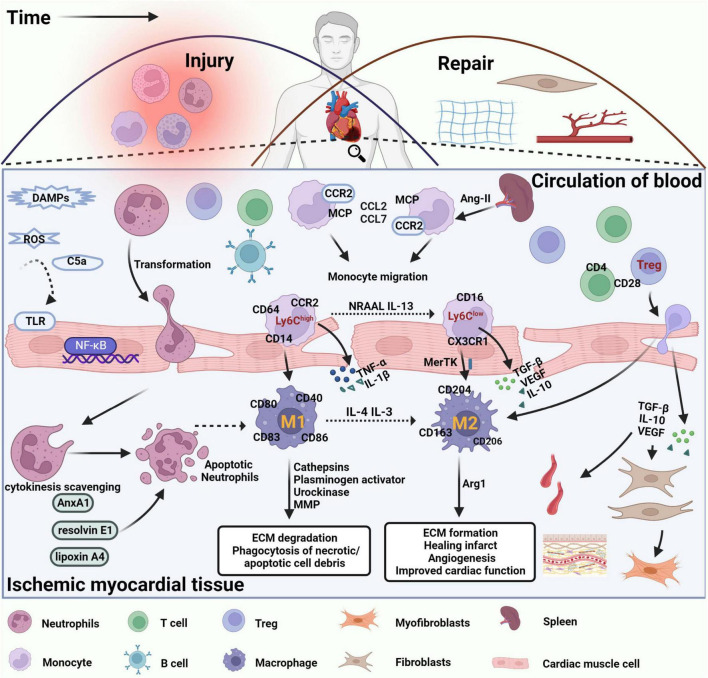
Immune cells are involved in damage and repair after myocardial infarction. After MI, DAMPs are released by necrotic cardiomyocytes and damaged extracellular matrix fragments, which activate TLRs and NF-κB signaling in cardiomyocytes, promote the release of inflammatory factors and chemokines, and leukocyte infiltration into damaged tissues. The inflammatory response is triggered by the disruption of antioxidant mechanisms, massive release of ROS, and significant activation of the complement system. Neutrophils are recruited into the infarct site within the first few minutes after MI, interact with endothelial cells via integrins and ICAM-1, perform phagocytosis of necrotic cell debris, and initiate an apoptotic program with corresponding changes on the cell surface to promote cytokine clearance and eventual clearance by macrophages. Circulating lipoxin A4, resolvin E1, and AnxA1 induce neutrophil apoptosis and stimulate macrophage polarization toward an anti-inflammatory phenotype. Apoptotic neutrophils release alpha-defensins, a cascade of proteins that increases macrophage phagocytic activity and inhibits their ability to release inflammatory factors. Therefore, clearance of apoptotic neutrophils is considered the beginning of cardiac repair after MI. In the following hours, B and T lymphocytes are also recruited to the damaged site, promoting the recruitment of circulating monocytes to the area of infarction. Monocytes in the spleen, regulated by ANG II, also migrated to the location of myocardial injury, driving CCR2-dependent monocyte migration via MCP-1 binding to CCR2 and promoting the expression of CCL2 and CCL7. Pro-inflammatory Ly6C^high^ monocytes differentiate into proinflammatory macrophages (M1), which release inflammatory factors such as IL-1, TNF-α, and IL-12, exert phagocytosis and secrete protein hydrolases to promote digestion of tissue and clearance of necrotic debris in the infarcted region. Over time, reparative Ly6C^low^ monocytes become the predominant subtype and differentiate into anti-inflammatory macrophages (M2) that express pro-repair mediators such as IL-10, VEGF, and TGF-β1 to suppress the inflammatory response, promote myofibroblast proliferation, angiogenesis, and collagen fiber deposition, and promote cardiac repair after MI. In addition, regulatory T cells regulate macrophage phenotype through secretion of IL-10, VEGF, TGF-β1, or contact-dependent pathways to facilitate the regression of cardiac inflammation and improve cardiac function after MI.

Neutrophils are first recruited to the damaged site 6 to 24 h after MI. At 48–72 h following the infarction, pro-inflammatory monocytes and macrophages accumulate at the infarct site, contributing to myocardial cell death and injury (9, 10). As the inflammatory response intensifies, necrotic cells and extracellular matrix debris are mostly removed. At 4–7 days, anti-inflammatory monocytes, macrophages, and regulatory T cells (Treg) are recruited to the ischemia site to scar and neointima formation, replace granulation tissue containing new capillaries, nerves, and fibroblasts, and suppress the initial inflammatory response to promote cardiac repair after MI (Figure 1). The inflammatory response is a prerequisite for late cardiac repair. The current clinical application of anti-inflammatory strategies to reduce or eliminate the inflammatory response in patients with MI has not yielded good results (9). In addition, extensive anti-inflammatory interventions may have harmful effects. Therefore, anti-inflammatory strategies should focus on adjusting the balance between pro-inflammatory and repair cell subpopulations. Enhances intrinsic anti-inflammatory effects or inhibits pro-inflammatory pathways, reducing the deleterious effects of the inflammatory response and preserving the beneficial effects during cardiac repair after MI (9, 10).

This article discusses the immune cells coordinating the inflammatory response following a MI, including neutrophils, monocytes, macrophages, and Treg. We explored how epigenetic modifications interfered with the inflammatory response and regulation of immune cells after MI and enumerated mice some potential therapeutic targets mediating cardiac repair after MI, aiming to provide new ideas for lightening myocardial injury after MI.

## Neutrophils

Neutrophils are transcriptionally active, complex cells capable of altering membrane molecule expression and producing inflammatory cytokines in response to inflammatory stimuli (11, 12). Furthermore, neutrophil apoptosis can regulate macrophage polarization for long-term immune responses (13, 14). Recent studies have constructed engineered neutrophil apoptotic bodies (eNABs) to mimic natural neutrophil apoptosis. eNABs actively target macrophages and can initiate the heme biosynthesis pathway to produce anti-inflammatory bilirubin, modulate the inflammatory response and enhance cardiac repair after MI (15). The heterogeneity and plasticity of neutrophils are also important in regulating the inflammatory response and promoting cardiac repair after MI (16).

### Role played by heterogeneity and plasticity in cardiac repair

Neutrophils can be classified into N1 (Ly6G^+^ CD206^–^) and N2 (Ly6G^+^ CD206^+^) phenotypes based on the type of cytokine production, the potential for macrophage activation, and surface molecule expression (16). N1 cells highly expressed pro-inflammatory markers such as IL-1β, IL-12a, TNF-α, classically activated macrophages, and expressed CD49d^high^ CD11b^low^, whereas N2 cells highly expressed anti-inflammatory attributes such as IL-10 or activated macrophages, and expressed CD49d^low^ CD11b^high^ (16). The heterogeneity of neutrophils isolated from the left ventricular (LV) after MI was further demonstrated by gene expression profiling and flow cytometry analysis of neutrophils (17). The MI mice model found that although CD206^–^ N1 neutrophil numbers consistently dominated the first 7 days after MI, CD206^+^ N2 neutrophils gradually increased 5 to 7 days after MI (18, 19). Furthermore, CD206^+^ N2 neutrophils were not found in the peripheral blood of MI mice, suggesting that N2 neutrophils are locally formed in the ischemic cardiac microenvironment. This is consistent with previous reports that the ischemic cardiac microenvironment is sufficient to promote CD206 expression on resident macrophages without IL-4 signaling (19). *In vitro* studies have shown that interferon-gamma (INF-γ) plus lipopolysaccharide (LPS) induces the N1 phenotype and IL-4 induction induces the N2 phenotype. When exogenous IL-4 is continuously infused 24 h after MI, proinflammatory cytokines are reduced in neutrophils, which implies a conversion of neutrophils to a repair phenotype (20). Nevertheless, the underlying mechanisms of the Ly6G^+^ CD206^+^ subpopulation in the post-MI phase of inflammatory regression remain unclear. To determine the possibility of whether Ly6G^+^ CD206^+^ neutrophils have anti-inflammatory activity, pro-angiogenic, and pro-fibrotic effects, this subpopulation was identified under conditions of induced angiogenesis in transplanted hypoxic tissue. Gene expression analysis demonstrates that this subpopulation of neutrophils has low pro-inflammatory activity and high anti-inflammatory and remodeling activity (21, 22). By correlating N1 and N2 neutrophils with LV functional variables, it was found that LV infarct wall thinning was positively correlated with N1. This may be associated with increased expression of MMP-12 and MMP-25. And LV infarct wall thinning was negatively correlated with N2 neutrophil polarization (17). Moreover, neutrophils can differentiate into other myeloid cell types in an inflammatory environment. Combining neutrophils with discrete cytokines and incubating for long periods can result in antigen-like presentation properties and dendritic cell (DC) characteristics or reprogramming to macrophages (23–26). The neutrophil’s role in injury and repair after MI may be explained in this way.

### Effect of clearance of apoptotic neutrophils on cardiac repair

After MI, the adhesion of integrins to cellular debris increased the activation of calcium and calpain in neutrophils and the size of neutrophil membranes, promoting the function of neutrophils in the phagocytosis of cellular debris (27–29). After completing their phagocytosis, neutrophils must be promptly removed by apoptosis or other means of death. The persistent presence of neutrophils leads to tissue damage and chronic inflammation. Delayed clearance of apoptotic neutrophils is associated with multiple human inflammatory diseases (30). Available studies demonstrate neutrophils contribute to ischemia/reperfusion (I/R) injury. In the ischemic border zone, neutrophils produce and release ROS, leading to acute inflammation and myocardial apoptosis (31). Ultimately, reperfusion damages the originally ischemic myocardium and exacerbates the inflammatory response, causing further impairment of cardiac function and tissue (32). Apoptosis of neutrophils results in corresponding changes on the cell surface, inducing changes in the surface charge of lipids and amino sugars by exposing intracellular molecules such as phosphatidylserine and calreticulin, promoting the exocytosis effect of macrophages (30, 33). In necrotic myocardial tissue, apoptotic neutrophils are predominantly cleared by macrophages, with a small proportion passing through DC to draining lymph nodes (33). In others, a portion of apoptotic neutrophils migrates via the endothelium by reverse migration from the necrotic myocardial tissue into the vessel’s lumen (34).

Clearing apoptotic neutrophils are considered the beginning of inflammation regression and repair after MI (35). First, apoptotic neutrophils can remove cytokines and chemokines to limit their recruitment to the infarcted area. After being phagocytosed by macrophages, it promotes the production of anti-inflammatory factors such as transforming growth factor-β (TGF-β), IL-10, vascular endothelial growth factor (VEGF), and the specialized pro-catabolic mediator SMP to reduce the inflammatory response (36–38). Second, dead or necrotic neutrophils release antimicrobial alpha-defensins that increase and activate macrophage phagocytosis and inhibit inflammatory cytokine release (39). In others, circulating platelets induce neutrophil apoptosis and anti-inflammatory polarization of macrophages through the release of abundant pro-resolution mediators, such as lipotoxin A4, lipolytic E1, and annexin A1 (AnxA1), thereby promoting the regression of inflammation (40–42). Based on a related MI model, inhibition of MMP-12 reduces the expression of CD44, caspase 3, and caspase 8 to inhibit neutrophil apoptosis, leading to a prolonged inflammatory response and further deterioration of LV function (43).

Neutrophil extracellular traps (NETs) are chromatin-based reticular structures, histones, and particles with protein hydrolases that form in the vascular system in infectious and non-infectious diseases. NETs induce monocytes to differentiate into intermediate subpopulations, promoting fibrin deposition and fibrin networks leading to thrombosis (44, 45). Therefore, the magnitude of the intensity of NETs formation directly affects the size of the MI infarct area. In peptide arginine deiminase 4 (PAD4) deficient mice, reduced NET production or increased NET degradation reduced neutrophil infiltration, reduced infarct size, and improved cardiac function (46). Because the NET formation process differs from necrosis or apoptosis, this novel form of neutrophil death *in vivo* is known as NETosis. Macrophages can also clear NETosis, and the uptake of NETs by macrophages does not induce the secretion of pro-inflammatory cytokines, which may be a potential mechanism by which macrophages can promote the regression of inflammation (47).

### Effect of neutrophil-mediated crosstalk on cardiac repair

Post-MI neutrophils can also promote inflammatory regression and cardiac repair by inducing reparative macrophages, promoting monocyte recruitment, promoting angiogenesis, regulating fibroblast function, and interacting with platelets.

The co-cultivation of neutrophils with activated macrophages inhibits the NF-κB pathway and reduces macrophage-released pro-inflammatory factors (48). The study found that the expression of phagocytic receptor Tyrosine-protein kinase Mer (MerTK), a primary apoptotic cell receptor on macrophages, was reduced in neutrophil-depleted MI mice models, all demonstrating that neutrophils can promote cardiac repair after MI by inducing reparative macrophages (49). In addition, depletion of neutrophils in wild-type mice with anti-Ly6G monoclonal antibody results in reduced Ly6C^high^ release from the spleen of wild-type mice and failure of timely clearance of necrotic cells and tissue debris, leading to impaired cardiac repair. Neutrophils can also improve cardiac repair after MI by promoting neovascularization through direct or induced macrophage production of VEGF-A, which provides nutrients and oxygen to the tissues surrounding the infarcted area to limit the expansion of the infarcted area. Inhibition of CD49d^+^ VEGFR1^high^ CXCR4^high^ neutrophil subpopulation was recently found to impair vascular neogenesis in humans and mice (21, 50).

Neutrophils can interact with fibroblasts to have an impact on cardiac repair. TGF-β promotes the differentiation of cardiac fibroblasts into myofibroblasts after MI by regulating the Smad3 signaling pathway and secretes collagen and other ECM proteins to repair myocardial tissue and temporarily maintain the structure and function of the cardiac (51). Fibroblasts with Smad3 or TGFbR1/2 genes transformed into myofibroblasts upon TGF-β stimulation after infection with adenovirus expressing beta-gal, marked by α-SMA expression. In contrast, fibroblasts infected with Cre’s adenovirus block the expression of TGFbR1/2 or Smad3, leading to impaired transdifferentiation of this cell and thus attenuating cardiac fibrosis and ECM remodeling (52). In a mice model of stress overload, daily injections of Anti-TGF-β neutralizing antibodies starting before surgery inhibited fibroblast activation (53). However, fibroblasts are significant players in the resolution of inflammation and scar formation after MI and are equally important for cardiac repair after MI (54). Whole transcriptome analysis of fibroblasts isolated from infarcted regions of C57BL/6J male mice by RNA sequencing revealed that on day 1 after MI, fibroblasts exhibited a pro-inflammatory phenotype contributing inflammatory cytokines to promote leukocyte recruitment to clear necrotic cardiomyocytes. On day 3 after MI, fibroblasts have anti-inflammatory, pro-fibrotic, and pro-angiogenic effects. On day 7 after MI, fibroblasts produce ECM and inhibit angiogenesis through Thbs1 signaling (54, 55). Co-culture of naïve neutrophils with cardiac fibroblasts *in vitro* upregulated TGF-β1 expression, further demonstrating that neutrophils can regulate fibroblast function to promote cardiac repair after MI (56).

Platelet-neutrophil complexes (PNCs) are associated with the pathogenesis of myocardial I/R (41, 57, 58). After MI, platelets form PNCs by releasing inflammatory mediators to promote neutrophil recruitment, which further exacerbates the extent of myocardial injury (42). In experiments on male C57BL/6J mice with permanent occlusion of the left coronary artery, a time-dependent accumulation of platelets in the infarcted myocardium was found, leading to a local inflammatory response, ventricular remodeling, and rupture (59). In contrast, antiplatelet therapy inhibits the inflammatory response in the infarcted myocardium and reduces post-infarct ventricular rupture and remodeling (59). Vasodilator-stimulated phosphoprotein (VASP) modulates dynamic changes in the cytoskeleton, thereby regulating the adhesion between neutrophils and platelets (58, 60). Phosphorylation of VASP at Ser153 or Ser235 was observed to reduce the formation of intravascular PNCs and the presence of PNCs in ischemic myocardial tissue in a model of myocardial I/R injury, resulting in a significant reduction in MI size (58). Thus, in addition to mediating ischemic injury after MI, platelets influence inflammatory cell infiltration and negotiate cardiac repair after MI.

Although many trials have shown that antineutrophil therapy can reduce cardiac injury in the acute phase, this has not been the case when applied to the clinical setting (61). The pro-repair effects of neutrophils should be considered when designing anti-inflammatory approaches because of the multifaceted functions of neutrophils. Any therapeutic strategy targeting neutrophils must strike a good balance between reducing the pro-inflammatory effect and maintaining the pro-repair development of neutrophils.

## Monocytes

After MI, circulating monocytes are first recruited to the damaged myocardium in response to stimulation by environmental factors and subsequently differentiate into various types of macrophages or DCs, or secrete large amounts of cytokines to mediate the development or regression of inflammation (62). Monocytes stored in the spleen are distinct from circulating monocytes. In response to angiotensin II (Ang II) signaling during MI, all splenic monocytes are mobilized and flow into the myocardial tissue to participate in the post-infarction inflammatory and repair process. Splenectomy experiments have shown that half of the mononuclear cells in the infarcted cardiac originate from the spleen (63).

Based on the expression of CD14 (lipopolysaccharide receptor) and CD16 (FcγRIII immunoglobulin receptor), human monocytes were classified into classic monocytes (CD14^++^ CD16^–^) and atypical (CD14^+^ CD16^++^) monocytes (64). Studies show significant similarities between human and mice monocyte subpopulations (65). Classic monocytes correspond to mice Ly6C^high^ monocytes, which account for approximately 80% to 85% of peripheral circulating blood monocytes and highly express CD14, CCR2, CD62L, and CD64. Atypical monocytes correspond to mice Ly6C^low^ cells, which account for a smaller proportion and highly express CD16, and CX3CR1 does not express CCR2 (65). Two stages are involved in the recruitment of circulating monocytes from the bone marrow and spleen to the infarcted region after MI. The first stage is dominated by the classical monocyte subpopulation, which peaks on day 3 after MI and can produce inflammatory cytokines (TNF-α, IL-1β), myeloperoxidase, and other inflammatory mediators. It can differentiate into macrophages and DCs, which promote the digestion of tissue in the infarcted area and the clearance of necrotic debris through phagocytosis and secretion of protein hydrolases (66). It is often described as inflammatory monocytes, even though existing studies have not proven that classical monocytes are precursors of macrophages (67). A key factor in the recruitment of monocytes to infarcted areas is the monocyte chemotactic protein-1 (MCP-1) or its receptor CCR2. In mice MI models, targeted blockade of CCR2 expression reduced Ly6C^high^ monocyte recruitment, attenuated inflammation in the infarcted area, increased left ventricular ejection fraction (LVEF), and decreased left ventricular end-diastolic volume (LVEDV), and inhibited LV remodeling after MI (68). CCL2 and CCL7 are ligands for CCR2, which may originate from B cells in the ischemic myocardium. The depletion of B cells in mice after MI inhibited the production of CCL7, decreased the recruitment of monocytes, reduced myocardial injury, and improved cardiac function (69). The second stage, 3–10 days after MI, was dominated by non-classical monocytes, Ly6C^low^ monocytes are recruited to damaged myocardial tissue by the chemokine Fractalkine1 (a novel chemokine of CX3C family) and secrete various pro-repair mediators such as IL-10, TGF-β1, and VEGF to promote myofibroblast proliferation, angiogenesis, and collagen fibril deposition, and play a role in inflammation regression (70). Despite the lack of direct evidence that non-classical monocytes become macrophages, Fractalkine-CX3CR1 signaling deficiency causes macrophages in epididymal white adipose tissue to gravitate toward inflammatory subpopulations and exacerbate the inflammatory response in adipose tissue (71). Non-classical monocyte differentiation is closely regulated by the NR4A1 transcription factor. Mice deficiency of the NR4A1 gene causes Ly6C^high^ monocytes to express higher levels of CCR2 and differentiate into abnormal inflammatory macrophages, leading to impaired myocardial tissue healing and cardiac dysfunction (72). Fibroblasts also affect the recruitment and differentiation of monocytes (73). When monocytes were co-cultured with LPS-stimulated fibroblasts, the secretion of pro-inflammatory factors such as MCP-1, TNF-α, and IL-12 was significantly increased, and monocytes were enhanced in recruitment and more likely to differentiate into M1-type macrophages (73). In contrast, monocytes exhibited opposite results when co-cultured with TGF-β1-stimulated fibroblasts (73).

In conclusion, monocytes are critical regulators of injury and repair after MI. Ly6C^high^ monocytes are pro-inflammatory and predominate in the first phase after MI, promoting tissue clearance from the infarcted area. Ly6C^low^ is reparative and dominates during the remission of inflammation. Reducing the recruitment of Ly6C^high^ monocytes in the first phase would be detrimental to the body’s clearance of necrotic myocardial tissue and debris, but inhibiting the recruitment of Ly6C^low^ monocytes in the second phase would reduce microvascular neovascularization and collagen deposition (74). However, the rapid clearance of mononuclear phagocytes diminishes the immunomodulatory effects of mesenchymal stem cell (MSC)-derived extracellular vesicles (EVs) with anti-apoptotic and anti-inflammatory properties as the inflammatory response subsides (75). Regulation of monocyte recruitment time or subgroup ratios may benefit cardiac repair after MI.

## Macrophages

Different macrophage subpopulations play different roles in cardiac self-renewal, inflammatory response, injury repair, and remodeling. Regulating the function of macrophage subsets after MI can promote the healing and repair of damaged tissues. Macrophages in the cardiac and differentiating from circulating monocytes, are partially derived from the embryonic yolk sac (YS) and are referred to as resident macrophages. They colonize the cardiac during the embryonic period and maintain a stable cell population by self-replication under steady-state conditions, becoming the largest subpopulation of cardiac macrophages (76–78). During the first 3 days after MI, infarction sites are flooded with millions of neutrophils and monocytes that further accelerate inflammation. Stimulated by the surrounding environment, these monocytes express large amounts of Ly6C on their surface to differentiate into macrophages with high phagocytic and protein hydrolytic activity and produce inflammatory factors such as IL-1, IL-6, and TNF-α, gradually replacing macrophages of embryonic origin (79–81). However, Gata6 luminal macrophages located in the pericardial cavity are minimally invasive in the infarcted region after MI. Still, they cannot prevent cardiac fibrosis and repair the damaged cardiac (82).

### Effect of different macrophages on cardiac repair

Macrophages exhibit a high degree of plasticity and adaptability *in vivo* and *in vitro*. Macrophages evolve into different phenotypes according to the environment in which it lives. The M1 and M2 phenotypes are classified according to surface markers, function and cytokines produced. M1 macrophages are classically activated or pro-inflammatory macrophages that mainly secrete pro-inflammatory factors to promote the development of inflammation and participate in the clearance of pathogens (83). The M2 macrophages are alternatively activated or anti-inflammatory macrophages that stimulate tissue repair and regeneration and have pro-fibrotic and pro-angiogenic effects (84). After MI, the phenotypic profile of macrophages changes significantly. After cardiomyocyte death, myocardial tissue at the infarct site was rapidly infiltrated by Ly6C^high^ monocytes, peaking at approximately day 3 after MI. Ly6C^high^ monocytes differentiate into M1 macrophages under the stimulation of IFN-γ, TNF-α and secrete pro-inflammatory and chemokines (IL-12, IL-23, IL-27, TNF, CXCL9, CXCL10, and CXCL11) to exacerbate the inflammatory response while performing the functions of phagocytosis of apoptotic and necrotic cell debris and degradation of ECM (85). It has also been shown that M1-like macrophages can secrete pro-inflammatory miRNAs and pro-inflammatory exosomes to exacerbate myocardial injury after MI and inhibit cardiac healing by exerting anti-angiogenic effects (86). On days 4–7 after MI, Ly6C^low^ monocytes are preferentially recruited to damaged tissues and differentiate into M2 macrophages with the induction of IL-4 and IL-13. They highly express IL-10, IL-1, CD206, decoy type II receptors, scavenger, and galactose-type receptors. They secrete anti-inflammatory mediators such as CCL17, CCL22, and CCL24, promote the release of Arginase-1 (Arg1), and exert anti-inflammatory effects while initiating ECM remodeling and promoting angiogenesis to participate in tissue repair (86).

However, the origin of M1 and M2 macrophages or whether M2 macrophages are polarized from M1 macrophages remains controversial (87, 88). Some related studies have demonstrated that stimulation of M1 macrophages to the M2 phenotype polarization can reduce poor LV remodeling after MI, thereby improving cardiac function. M2 macrophages secrete cytokines (IL-10 and TGF-β) that initiate tissue remodeling and angiogenesis to inhibit the pro-inflammatory effects of M1 macrophages. The Trib1 gene is associated with the ability to form M2 macrophages. Trib1^–/–^ mice exhibit impaired fibroblast activation and reduced collagen synthesis in the infarcted region after MI, ultimately leading to frequent cardiac ruptures and a poor prognosis. Inputting exogenous M2 macrophages can rescue this phenomenon and promote myocardial tissue repair after MI (89). IL-4 and IL-10 have been shown to induce the M2 phenotype in infarcted cardiac (90, 91). The administration of long-acting IL-4 complex to mice after coronary artery ligation restored cardiac function, improved cardiac repair, and attenuated adverse ventricular remodeling. However, this effect was not observed in mice unable to form M2 macrophages, which was attributed to the fact that the repairing result of the cardiac was due to M2 macrophage activity rather than IL-4 action (90). The administration of exogenous IL-10 to C57BL/6J mice significantly reduced inflammation in the LV, and a significant increase in gene expression of M2 markers (Arg1, Ym1, and Tgfb5) was observed in the isolated macrophages (91).

As previously mentioned, the level of MHC-II^low^CCR2-macrophages in the YS decreases with age and is replaced by macrophages evolving from circulating monocytes after MI. However, these resident macrophages partially resemble the M2 macrophages phenotype and show cardioprotective properties after MI (89). By reducing resident macrophages, the peri-infarct region undergoes adverse remodeling, and cardiac function is impaired (92). The Lgmn gene is associated with the ability of resident cardiac macrophages to clear apoptotic cardiomyocytes in an MI mice model. Lgmn deficiency exacerbates myocardial injury by promoting the accumulation of apoptotic cardiomyocytes and reducing the index of effluent cells *in vivo*, and overexpression of Lgmn in cardiac macrophages using an adenoviral vector improves cardiac function in mice after MI (93). Thus, stimulating the generation of resident cardiac macrophages is a cell-mediated approach to cardiac repair after MI.

### Effect of macrophage-mediated crosstalk on cardiac repair

The anti-inflammatory and tissue repair effects of M2 macrophages are associated with fibroblast activation (91). Activated fibroblasts produce large amounts of prostaglandin E2 (PGE2) to inhibit TNF-α production by macrophages, thereby promoting macrophage phagocytosis of apoptotic neutrophils to mediate the regression of inflammation (94). IL-10-stimulated macrophages exhibited higher proliferation and migration rates. M2 macrophages can increase fibroblast proliferation to reduce the ratio of collagen I and III, thereby significantly decreasing myocardial fibrosis after MI (91, 95). Activated fibroblasts generate contractile force to reorganize ECM to prevent cardiac dilation and rupture after MI (91). Immune-mediated activation of cardiac fibrosis is necessary to avoid cardiac rupture after MI. However, in many cases, the initial beneficial fibroblast activation is uncontrolled, resulting in excessive ECM accumulation that hardens myocardial tissue and deteriorates cardiac function, thereby detrimental to cardiac repair.

M2 anti-inflammatory macrophages can also enhance the ability of mesenchymal stem cells (MSCs) in tissue repair, while MSCs can improve tissue repair by acting on macrophages (96). Animal models have shown this phenomenon. In a mice MI model, intravenous transplantation of human umbilical cord blood-derived mesenchymal stem cells (hUCB-MSCs) stimulated intra- cardiac and extra-cardiac macrophage M2 expression, reduced serum IL-6 and galectin-3 levels to decrease inflammatory responses and improved cardiac function (97). In contrast, the depletion of macrophages with clodronate liposomes attenuated the therapeutic effect of MSCs for MI, with increased infarct size, decreased ejection fraction, accelerated LV remodeling, and increased 30-day mortality in mice (98).

Extracellular vesicle mediates the cardioprotective effects of MSCs through immunomodulatory and anti-inflammatory effects. Targeting MSC-EVs to pro-inflammatory macrophages using engineering techniques can promote local myocardial immune regulation, thereby enhancing inflammation regression and cardiac repair (99, 100). Platelet binding to circulating monocytes is enhanced after MI/R, and the introduction of platelet membrane engineering (P-EVs) also enhances the targeted delivery of EVs to pro-inflammatory macrophages in the local myocardium (59, 101). In a mice model of MI/R injury, P-EVs bind to circulating Ly6C^high^ monocytes into ischemic myocardial tissue and preferentially differentiate into M1 macrophages after endothelial migration. P-EVs are then endocytosed *in situ* by M1 macrophages and escape from macrophage lysosomes, releasing miRNAs into the cytoplasm to amplify immunomodulatory effects and promote M1 macrophage polarization into M2 macrophages, ultimately promoting cardiac repair (101).

Adipose-derived stem cell (ADSC) exosomes could also promote macrophage M2 polarization via the S1P/SK1/S1PR1 signaling pathway in an animal model of MI, reducing the inflammatory response to improve cardiac injury after MI (102). Intracoronary infusion of cardiosphere-derived cells (CDC) 48 h after MI in pigs reduces infarct size and decreases microvascular occlusion while promoting the conversion of macrophages to a cardioprotective phenotype (103). In addition to reducing inflammatory myocardial injury, cardiac progenitor cells can promote cardiac repair by regulating monocyte/macrophage phenotypic changes. Cardiac progenitor cell transplantation is a desirable and promising therapeutic measure for MI.

In summary, macrophages play a dual role in the entire pathological process of MI. Among them, the anti-inflammatory and reparative effects of M2 macrophages after myocardial infarction have been recognized. Restoration of cardiac function after MI by M2 macrophages is mainly reflected in three aspects: inhibition of inflammatory response, promotion of angiogenesis and collagen formation, and regulation of macrophage polarization is the key to cardiac repair. Therefore, targeting macrophages offers a potential therapeutic opportunity for cardiac repair after MI.

## T cells

T cells can be divided into CD4^+^ T cells and CD8^+^ T cells according to their function and molecular phenotype. It is well known that CD4^+^ T cells are among the most functionally complex immune cells and can be polarized into different functional cell subpopulations by specific cytokines (104). Among them, a fraction of CD4^+^ T cells (Th1, Th2, and Th17) are mainly involved in the synthesis and secretion of cytokines and play a complementary and regulatory role in the immune response. Treg cells formed by TGF-β stimulation have immunosuppressive functions that downregulate the immune response and repair myocardial tissue in MI, mainly by suppressing the inflammatory response and activating fibrosis (105). The study of autopsy specimens from patients with MI revealed T-cell infiltration in the distal and peri-infarct areas of the MI region and the presence of activated T cells within the walls of both infarcted and non-infarcted coronary arteries (106). Tregs bio nanoparticles (CsA@PPTK) were recently prepared by camouflaging nanoparticles with platelet membranes. It allows effective drug delivery to the ischemic myocardial tissue, which may be a new strategy to improve cardiac function after MI/RI. CsA@PPTK scavenges ROS, increases Tregs production and M2/M1 ratio in the infarct area, and reduces infarct size and fibrosis area (107). Several studies have shown that CD4^+^ T cells cause myocardial I/R injury. When examining the infarct size after transient coronary artery occlusion in T-lymphocyte-deficient mice (RAG1-KO) and wild-type (WT) mice, it was found that the infarct size in RAG1-KO mice was significantly smaller than that in WT mice (108, 109). IFN-γ and IL-17 play an important role in this process. They amplify the death of cardiac cells and stimulate the proliferation of fibroblasts. Lack of IL-17A or γδ-T cells (the primary producer of IL-17 in the cardiac) reduced fibrosis and further infarct size expansion in non-infarcted myocardium (109). It has also been shown that CD4 knockout or MHC-II knockout in MI mice lacking a functional CD4^+^ T cell response results in increased LV expansion and higher total leukocyte and pro-inflammatory monocyte counts in myocardial tissue in the infarcted region in these mice compared to WT mice (110). Tregs are another CD4^+^ T lymphocyte subpopulation that is an essential cell type for myocardial repair and are the leading cause of this paradoxical outcome. Healthy myocardium has few resident Treg cells, but these cells can be recruited to infarct areas within hours of MI and persist for at least 1 week after that (111). The infarcted region of a rat MI model was found to be infiltrated with Treg cells. Treg cells were expanded *in vivo* by over-transfer and CD28 super-excited antibodies, ultimately improving cardiac function (112). To determine how Treg cells contribute to post-MI healing, the use of genetic Treg cells to ablate the MI mice mode revealed increased cardiac inflammation and worsened clinical outcomes. However, treatment with CD28 super-agonistic antibodies after MI resulted in Treg cell activation and increased collagen expression from scratch, reversing the above results (113, 114). Therefore, Tregs are necessary for post-MI repair. Due to their absence, the infarct size increases, inflammation occurs more intensely, and collagen deposition is impeded, resulting in poor cardiac remodeling (114). The beneficial effect of Treg cells on post-MI repair may be related to their ability to induce macrophage polarization toward M2 in the infarcted area and to produce TGF-β for myofibroblast activation (114, 115). Interestingly, fibroblasts can also interact with Treg to regulate the inflammatory response. When co-cultured with isolated cardiac fibroblasts, Tregs reduced the expression of MMP-3 and myofibroblast marker α-SMA, attenuated fibroblast matrix degradation activity, and avoided adverse cardiac remodeling (113). Tregs require intact CD39 (ectonucleoside diphosphate hydrolase-1) signaling for their beneficial effects, suggesting that controlling purinergic metabolism can help treat cardiovascular disease (116). Further studies showed that mice lacking CD73, another ectonucleotidase that converts AMP to adenosine, have impaired cardiac function after I/R (117). In addition, Treg can also improve cardiac tissue remodeling by secreting six major factors (Cst7, Tnfsf11, Il33, Fgl2, Matn2, and Igf2) in a paracrine manner to stimulate cardiomyocyte proliferation directly (111). All this evidence suggests that Tregs can inhibit the local inflammatory response after MI and promote the pro-fibrotic function of mesenchymal stromal cells through multiple mechanisms, benefiting cardiac repair after MI.

Cardiovascular repair after MI has also been associated with cytotoxic CD8^+^ T lymphocytes. Mice lacking CD8^+^ T lymphocytes delay clearance of necrotic tissue after MI and eventually die from myocardial rupture due to poor cardiac remodeling caused by dysfunctional fibrosis and increased inflammatory response (118). The infusion of CD8^+^ AT2R^+^ T cells into the myocardium of rats following MI results in a decrease in infarct size and an extended survival cycle, demonstrating the beneficial role of CD8^+^ T cells in the repair of the myocardium (119). Thus T cell-centered adaptive immunity is associated with cardiac remodeling after MI ischemia and plays an essential role in mediating tissue repair after cardiac injury.

## Epigenetic modifications as emerging targets for regulating inflammatory responses

There is strong evidence that genetic, epigenetic, and environmental factors interact to cause and progress MI, with epigenetics accelerating the onset and progression of MI. Epigenetic regulatory processes include multiple molecular mechanisms such as DNA methylation (DNAm), histone post-translational modifications, and RNA-based mechanisms (120). The evidence is mounting that epigenetic changes regulate inflammatory responses and immune cell phenotypes following MI, thereby responding to stimuli from environmental factors. Thus, it is possible to repair the cardiac after MI by targeting epigenetic mechanisms.

### Epigenetic modifications regulate the inflammatory response after myocardial infarction

DNA methylation (DNAm) alters the transcription process by adding methyl groups to specific DNA nucleotides, resulting in inactive gene expression. Myocardial ischemia is protected by aldehyde dehydrogenase 2 (ALDH2). Myocardial ischemic injury caused by the downregulation of ALDH2 after MI was associated with aberrant hypermethylation of the CpG site in the upstream sequence of the ALDH2 promoter, and myocardial injury after MI could be alleviated by enhanced activation of ALDH2 (121).

Histone modifications are mainly post-transcriptional and are the primary mechanism of epigenetic regulation. Phosphorylation, acetylation, methylation, and ubiquitination are the most common modalities (122). Histone deacetylase (HDAC) is a transcriptional regulator, and basic experiments have shown that HDAC inhibitors prevent cardiac remodeling after MI by relying on recovering cardiac fibroblast autophagosomes (123). In addition, HDAC inhibitors may promote cardiac repair and neovascularization in MI. Trazodactin A (TSA) is an effective HDAC inhibitor. Ventricular function was restored in TSA-treated wild-type Kit(+ / +) mice compared with Kit(W)/Kit(W-v) mice. Myocardial function and cardiac repair were restored and promoted by reintroducing TSA-treated wild-type C-kit(+) CSCs into Kit(W)/Kit(W-v) mice with MI. To further validate the beneficial effects of HDAC inhibitors, pretreatment of c-kit + CSC with HDAC by TSA significantly increased c-kit + CSC-derived myocytes and microvessels (124). It is unclear, however, whether specific HDAC4 is involved in the benefits of HDAC inhibition in promoting myocardial repair and maintaining cardiac function. It has even been shown that inhibition of HDAC reduces TNF-α in myocardium and serum, decreases ROS production, avoids cell death, and promotes cell viability, thereby improving recovery of myocardial function after MI (125, 126). TSA can also block α-SMA expression in fibroblasts and reduce AKT activation, thus inhibiting TGF-β1 from promoting fibroblast to myofibroblast transdifferentiation and avoiding adverse cardiac remodeling due to continuous activation of myofibroblasts (127).

In general, non-coding RNAs (ncRNAs) do not encode proteins but function as regulators of protein expression. These RNAs mainly consist of long non-coding RNAs (lncRNAs), microRNAs (miRNAs), small interfering RNAs (siRNAs), and circulating RNAs. A growing body of research shows that ncRNA plays a crucial role in MI and can be used to diagnose and treat MI as a potential biomarker and intervention target (128, 129). MiR-214 is a newly discovered miRNA that can inhibit apoptosis in cardiomyocytes. In the MI rat model, by overexpressing miR-214, infarct size was reduced, cardiac function and hemodynamic status were improved, and LV remodeling was inhibited (130). It has been demonstrated that some miRNAs are essential in reducing the inflammatory response after MI. MiR-155 overexpression increased inflammation and leukocyte infiltration in a mice model of MI. At the same time, this effect was eliminated by knocking down the RNA of SOCS-1, the direct target gene of miR-155 (131). MiR-146a is the miRNA most abundant in CDC exosomes. It reduces inflammation after MI by inhibiting the expression of Irak1 and Traf6. Larger scar masses and thicker infarct wall thickness were observed in MI mice knocked out of miR-146a compared to wild-type mice. Exogenous injection of miR-146a decreased the frequency of cardiomyocyte apoptosis and increased the live tissue of the cardiac (132). In addition, exogenous hydrogen sulfide can attenuate NLRP3 inflammatory vesicle and cysteine-1 formation in a miR-21-dependent manner to reduce the inflammatory response after MI (133). This suggests that cardiac repair after MI may be facilitated by targeting this inflammatory miRNA. NcRNA also regulates angiogenesis and fibrosis in the infarcted region after MI. MiR-126 promotes angiogenesis after MI by acting as a central regulator (134). In addition, plasma levels of miR-126 correlated with platelet activation (135). Overexpression of miR-126 was observed in an MI mice model to enhance functional myocardial angiogenesis in MI by upregulating VEGF, FGF (essential fibroblast growth factor), and DLL4 (notch ligand Δ-4 in mesenchymal stem cells) (136). Studies show that cardiac fibrosis is associated with the miR-34 family after MI and that silencing the entire miR-34 family protects MI mice from pathological cardiac remodeling and improves cardiac function via the TGF-β1/Smad4 pathway (137, 138).

Although the above evidence suggests that modulating epigenetic modifications can promote the regression of the inflammatory response and cardiac repair after MI, the exact mechanisms of how they act on post-MI inflammatory cells have not been elucidated.

### Epigenetic modifications regulate the phenotype of immune cells after myocardial infarction

Cardiac repair after MI requires neutrophils, and several new studies suggest that HDAC contributes to regulating neutrophil proliferation, differentiation, and apoptosis. Neutrophils in HDAC11-deficient mice exhibited more pro-inflammatory effects, higher migratory and phagocytic capacities, and higher TNF-α and IL-6 expression levels than wild-type mice (139). Therefore, targeting neutrophils by modulating histone modifications may benefit cardiac repair after MI.

Studies have shown that epigenetic modifications increase cardiac repair after MI by regulating monocyte/macrophage phenotypes and inflammation genes (140). Jmjd3, which demethylates H3K27, is essential for M2 polarization in macrophages of mice treated with IL-4. It induces Irf4, Arg1, CD206, and other M2-specific markers (141). Smad3 is an H3K4 methyltransferase that also regulates M2 polarization in macrophages, and its expression level in human monocyte-derived macrophages increases with stimulation of M-CSF, IL-4, and IL-13 (142). In general, inhibition of HDAC exerts anti-inflammatory effects, and monocytes and macrophages using HDAC inhibitors can reduce the production of inflammatory cytokines in an inflammatory environment (143, 144). It was shown that HDAC3 deficiency in macrophages leads to IL-4-induced expression of alternative activating genes and fails to activate most of the inflammatory gene expression program upon LPS stimulation, depending on the activation of the autocrine IFN-β/STAT1 circuit (145, 146). In addition, HDAC4 was also associated with anti-inflammatory effects, and in human monocyte-derived DCs, HDAC4 regulated STAT6 activity and anti-inflammatory gene expression (147). HDAC5 is associated with pro-inflammatory macrophages, and overexpression of HDAC5 in RAW264.7 cells significantly increased the activity of TNF-α, NF-κB, and the secretion of other inflammatory mediators (148). MiR-33 regulates macrophage metabolism, reduces fatty acid oxidation, and promotes glycolysis, thereby maintaining a pro-inflammatory M1 phenotype. Inhibition of miR-33 metabolism repolarizes macrophages to an M2 phenotype with anti-inflammatory and tissue repair effects and induces the accumulation of Foxp3(+) Tregs (149). Although recent studies suggest an interaction between RNA-based mechanisms and histone-modifying enzymes in epigenetic modifications, the exact mechanism in the monocytes/macrophages phenotype is unclear (150).

Transcription factor forkhead box protein 3 (Foxp3) is a master regulator of Treg function, and inhibition of methylation using 5-aza-29-deoxycytidine (DAC) or knockdown of DNA methyltransferase DNMT1 both induce Foxp3 expression and increase Treg number (151). *In vitro* stimulation of human Tregs results in CpG methylation of conserved regions within the Foxp3 locus in CD4^+^ CD25^+^ CD127^low^, leading to Foxp3 expression being lost and pro-inflammatory cytokines being produced (152). In addition, miR-155 is considered critical for maintaining Treg adaptability under adverse conditions. Foxp3-dependent miR-155 enhances the survival of Treg cells by activating STAT5 through the down-regulation of STAT pathway inhibitor SOCS1 protein and enhancing signaling to IL-2 (153).

Therefore, further work is needed to determine which epigenetic mechanisms play a role in neutrophils, monocytes, macrophages, and Treg cells so that these epigenetic modalities can be modulated to promote cardiac tissue repair and inflammation regression in the treatment of post-MI. Although modulating epigenetic modifications can effectively reduce infarct size and promote cardiac repair after MI, applying them to the clinic faces serious challenges. First, we do not fully understand the exact mechanisms of epigenetic modifications. Second, there is some use of vectors in gene therapy, and safety and efficacy still need to be thoroughly evaluated. Thus, the pathway of epigenetic modification has not yet reached the stage of translation to the clinic.

## Potential therapeutic targets mediating the inflammatory response and cardiac repair after myocardial infarction

To date, although some basic experiments have demonstrated that the inflammatory response is a significant event in the development of MI, it can have insignificant or detrimental effects in clinical trials applying anti-inflammatory strategies to treat MI. So there is a strong need to find some therapeutic targets linking the inflammatory response to the repair process after. S100A8/A9 is a pro-inflammatory alarmin that is released in significant quantities by necrotic cells and activated neutrophils at the infarct site after MI, enters the coronary arteries and body circulation before the rise of myocardial injury markers, and exacerbates the inflammatory response by binding to RAGE and TLR4 as DAMPs (154–156). Related experiments showed that short-term blockade of S100A9 with the specific blocker ABR-238901 in MI wild-type C57BL/6 mice during the inflammatory phase of the immune response reduced neutrophil and macrophage infiltration in MI, suppressed myocardial and systemic inflammatory responses, and facilitated the expression of repair proteins, thereby improving cardiac function (157). In contrast, recombinant S100A8/A9 treatment in the I/R mice model exacerbated myocardial injury and cardiac failure (158). In assessing whether S100A8/A9 can affect neutrophil phenotypic function, it was found that blocking S100A8/A9 reduced the migration rate of phosphorylation of ERK1/2 and p65 in N1 neutrophils, decreased the production of chemokines CCL2, CCL3, and CCL5, and attenuated the activity of NO, MPO, and MMP-9 (159). In addition, S100A8/A9 can induce NF-κB activation by promoting TNF-α secretion by macrophages and promoting MMP-9 expression by fibroblast cells, leading to cardiac rupture after MI (160). In a mice model of permanent MI occlusion, S100A9 is also a functional effector of LV infarct wall thinning (161). Based on the above evidence, S100A8/A9 may have therapeutic potential by linking inflammatory response and repair after MI.

Signaling pathways such as Wnt/Fzd play a unique role in inflammation, myocardial repair, and cardiac hypertrophy after MI. The Wnt/Fzd signaling pathway is ordinarily quiescent in the adult cardiac but is reactivated after MI to participate in the tissue repair process. Frizzled-related protein-1 (FrzA/sFRP-1), an antagonist of this pathway, reduces inflammation significantly, attenuates the progression of cardiac fibrosis, and benefits cardiac repair after MI (162). Transplantation of bone marrow cells (BMCs) from sFRP-1 transgenic mice into wild-type mice revealed better myocardial healing in wild-type mice transplanted with BMCs. sFRP-1 significantly reduced the infiltration of neutrophils in ischemic tissue, reduced the size of the post-infarction scar, and improved cardiac hemodynamic parameters (162). This phenomenon was also found in FrzA transgenic mice, with reduced cardiac rupture rate, reduced infarct size, improved collagen distribution, reduced apoptotic index, early leukocyte infiltration, and MMP-2 and MMP-9 activity after MI (163). However, it is worth noting that inhibition of the Wnt/Fzd signaling pathway presents a cardioprotective effect while blocking the Wnt/Fzd signaling pathway instead leads to an increase in the rate of cardiac rupture (163). In this way, it is possible to improve the prognosis after MI by overexpressing sFRP-1 via specific methods on time.

Podoplanin (PDPN) is a mucin-type transmembrane glycoprotein. Following a MI, the number of PDPN-positive cells increases gradually (164). In the MI animal model, the LV of animals treated with PDPN-neutralizing Ab showed better systolic and diastolic function after stimulation with epinephrine compared to controls (164). In addition, the co-culture of PDPN-positive cells with monocytes helps stimulate monocytes to produce pro-inflammatory cytokines and promote the recruitment of other inflammatory cells (164, 165). In contrast, using PDPN-neutralizing Ab enables an increase in therapeutic CD163 monocytes/macrophages and the expression of anti-inflammatory cytokines such as IL-10 (164). Assembly of NKX-2.5-positive cells and enhanced angiogenesis were also found in infarcted areas of animals treated with PDPN-neutralizing Ab. A myocyte transcription factor is expressed in the early stages of progenitor/stem cell differentiation (164). Thus, neutralizing PDPN may lead to a balance of pro-inflammatory and anti-inflammatory signaling through interaction with monocytes/macrophages to protect the cardiac from worsening ischemic injury, enhancing cardiac repair after MI and improving cardiac function (165).

## Conclusion

In summary, various molecular and cellular events occur during cardiac repair after MI. After the myocardial injury, poor remodeling is primarily caused by a worsening and prolonged inflammatory response. Targeted anti-inflammatory strategies have been extensively studied for improving cardiac repair after MI. Immune cells are indispensable in the inflammatory and repair phases following MI. Immune cells produce pro-inflammatory factors during the inflammatory response phase to perform the function of phagocytosis or removal of necrotic cardiomyocytes and cellular debris. During the proliferation and maturation phases, they produce anti-inflammatory cytokines to eliminate the inflammatory response, induce proliferation of cardiomyocytes and fibroblasts, and stimulate neovascularization and extracellular matrix production (166). Additionally, these immune cells amplify the inflammatory response or promote inflammation regression by interacting with platelets and fibroblasts, ultimately helping to remove necrotic cardiomyocytes and promote cardiac repair. An imbalance in the ratio of different immune cells or an imbalance in different subtypes of the same immune cells may adversely affect cardiac repair after MI. Therefore appropriate interventions to adjust immune cells to their normal physiological state may provide new therapeutic avenues for MI. Thus, an in-depth exploration of the mechanisms of immune cell interactions and immune cell interactions with platelets, fibroblasts, and other cells in the cardiac, revealing how epigenetic modifications regulate the balance of immune cells and target imbalanced immune responses within a specific time window, is crucial for intervention strategies for cardiac repair after MI that target inflammation (Tables 1, 2; 167).

**TABLE 1:** Experimental evidence for immune cell-mediated inflammation and cardiac repair.

	Intervention strategies	Inflammatory response	Cardiac effect	Quotations
Neutrophils	Inhibits MMP-12	Pro-inflammatory cytokines↑ Inflammatory response↑ Inhibition of neutrophil apoptosis	Deterioration of LV function	(43)
	Depleted neutrophils	Impaired inflammation subsides Insufficient monocyte recruitment MerTK expression↓	Deterioration of cardiac function Fibrosis↑ Cardiac failure occurs↑	(49)
	Inhibits CD49	Neutrophil count↓	Impaired angiogenesis	(50)
	Tetrandrine	Neutrophil infiltration↓ ROS↓	Myocardial infarction area↓ MI/R related deaths↓	(168)
	Anti-RANKL antibody	Neutrophil infiltration↓ ROS, MMP-9 release↓	Myocardial infarction area↓ Improved cardiac function	(169)
	Blocking S100A9	Neutrophil infiltration↓ Monocyte/macrophage count↓ Inflammatory response↓	Repair protein expression LVEF↑ Cardiac output↑ Improved cardiac function	(157)
Monocytes/macrophages	Blocking CCR2	Ly-6Chigh monocyte recruitment↓ Inflammation in the infarct area↓	LVEF↑ LVEDV↓	(68)
	VGSC inhibitor	Pro-inflammatory monocytes↓ M1 to M2 macrophage polarization↑	Late myocardial infarction size↓ LV fibrosis↓	(170)
	Knockout of Trib1 gene	Selective depletion of M2 macrophages↑	Collagen synthesis↓ Frequent cardiac ruptures	(89)
	Agonism of GABAA receptors	Pro-inflammatory monocytes↓ M1 to M2 macrophage differentiation↑	Late myocardial infarction volume↓ Unfavorable LV remodeling↓	(171)
	PDPN neutralizes Ab	Restorative CD163 monocyte/macrophage↑ Anti-inflammatory cytokines↑	Improved cardiac function	(165)
Treg	CD28 hyper-excitable antibody	TNF-α and IL-1β↓ IL-10↑	Collagen expression↑ Improved cardiac function	(112, 114)

**TABLE 2:** Experimental evidence that epigenetic modifications modulate inflammatory responses affecting cardiac repair.

	Intervention strategies	Inflammatory response	Cardiac effect	Quotations
DNA methylation	Overexpressing ALDH2	AMPK and Akt/mTOR signaling↑ Apoptosis↑	Myocardial injury↓	(121)
	DAC	Induction of FoxP3 expression Treg number↑	Improved cardiac function	(151)
Histone modification	TSA	Pro-inflammatory cytokines↓	Cardiac fibroblast autophagosome recovery↑ Undesirable cardiac remodeling↓ Neovascular formation↑	(123–126)
	Knockdown of HDAC11	Pro-inflammatory and phagocytic capacity of neutrophils↑ TNF-α, IL-6↑	Occurrence of septic shock↑ Cardiac inflammation↑	(139)
	M-CSF, IL-4 and IL-13 stimulation	M2 polarization↑ SMYD3↑	—	(142)
	Overexpression of HDAC5	TNF-α, NF-κB activity↑ Secretion of other inflammatory mediators↑	—	(148)
Non-coding RNA	Overexpression of miR-214	Inflammatory factor expression↓	Infarct area↓ Improved hemodynamic status LV reinvention↓ Improved cardiac function	(130)
	Overexpression of miR-155	Infiltration of leukocytes↑ TNF-α, IL-1b, Caspase3, CD105 expression↑	Myocardial infarction area↑	(131)
	Knockdown of miR-146a	Irak1 and Traf6↑	Keloid masses↑Proliferation of cardiomyocytes↓Angiogenesis↓ Infarct wall thickness↑	(132)
	Overexpression of miR-126	VEGF, FGF, DLL4↑	Myocardial functional angiogenesis↑	(136)

Until now, immunomodulatory anti-inflammatory drugs for MI have not been used in clinical practice. Most experimental models used to verify that anti-inflammatory strategies benefit cardiac repair are induced in young, healthy animals by blocking healthy coronary arteries. However, in clinical practice, most patients with MI suffer from an acute ischemic injury due to coronary atherosclerotic plaque rupture or thrombosis blocking the coronary arteries. Most patients with MI are middle-aged and elderly. Therefore, in future studies, the effects of age, comorbid diseases (diabetes, hypertension, hyperlipidemia, etc.), and adjuvant medications (statins, β-blockers) should be considered, using animal models of MI that are more clinically relevant.

In addition, related experiments have demonstrated that targeting components other than immune cells (including dendritic cells, mast cells, and cardiac progenitor cells) can also have beneficial effects on cardiac repair. Therefore future studies should also explore the inflammatory response and the interactions between these cells to promote cardiac repair after MI.

## Data availability statement

The original contributions presented in this study are included in the article/supplementary material, further inquiries can be directed to the corresponding author/s.

## Author contributions

TL, ZY, and YF conceptualized and wrote the manuscript. XF and AL assisted in drafting the manuscript. ZQ, ZY, and JZ revised the manuscript critically and provided important intellectual content. ZQ and JZ conceptualized the manuscript and guided the writing process. All authors have contributed significantly.
